# Temporal Ensemble Logic for Integrative Representation of the Entirety of Clinical Trials

**DOI:** 10.4230/LIPIcs.TIME.2025.13

**Published:** 2025-10-13

**Authors:** Xiaojin Li, Yan Huang, Rashmie Abeysinghe, Zenan Sun, Hongyu Chen, Pengze Li, Xing He, Shiqiang Tao, Cui Tao, Jiang Bian, Licong Cui, Guo-Qiang Zhang

**Affiliations:** The University of Texas Health Science Center at Houston, TX, USA; The University of Texas Health Science Center at Houston, TX, USA; The University of Texas Health Science Center at Houston, TX, USA; The University of Texas Health Science Center at Houston, TX, USA; University of Florida, Gainesville, FL, USA; Mayo Clinic in Florida, Jacksonville, FL, USA; Indiana University Bloomington, Bloomington, IN, USA; The University of Texas Health Science Center at Houston, TX, USA; Mayo Clinic in Florida, Jacksonville, FL, USA; Indiana University Bloomington, Bloomington, IN, USA; The University of Texas Health Science Center at Houston, TX, USA; The University of Texas Health Science Center at Houston, TX, USA

**Keywords:** Temporal ensemble logic, Clinical trials, Logic-based modeling, Theory of computation, Theory and algorithms for application domains

## Abstract

Clinical trials are typically specified with protocols that define eligibility criteria, treatment regimens, follow-up schedules, and outcome assessments. Temporality is a hallmark of all clinical trials, reflected within and across trial components, with complex dependencies unfolding across multiple time points. Despite their importance, clinical trial protocols are described in free-text format, limiting their semantic precision and the ability to support automated reasoning, leverage data across studies and sites, or simulate trial execution under varying assumptions using Real-World Data. This paper introduces a formalized representation of clinical trials using Temporal Ensemble Logic (TEL). TEL incorporates metricized modal operators, such as “always until *t*” (${◻}_{t}$) and “possibly until *t*” (${♢}_{t}$), where *t* is a time-length parameter, to offer a logical framework for capturing phenotypes in biomedicine. TEL is more expressive in syntax than classical linear temporal logic (LTL) while maintaining the simplicity of semantic structures. The attributes of TEL are exploited in this paper to formally represent not only individual clinical trial components, but also the timing and sequential dependencies of these components as a whole. Modeling strategies and demonstration case studies are provided to show that TEL can represent the entirety of clinical trials, whereby providing a formal logical framework that can be used to represent the intricate temporal dependencies in trial structure specification. Since clinical trials are a cornerstone of evidence-based medicine, serving as the scientific basis for evaluating the safety, efficacy, and comparative effectiveness of therapeutic interventions, results reported here can serve as a stepping stone that leads to scalable, consistent, and reproducible representation and simulation of clinical trials across all disease domains.

## 1 Introduction

Clinical trials are a cornerstone of biomedical research, providing rigorous evidence for the safety, efficacy, and comparative effectiveness of therapeutic interventions [32]. These studies follow structured protocols that define eligibility criteria, intervention timing, follow-up assessments, and outcome measures. Each of these components is inherently temporal, unfolding across defined intervals and often involving complex interdependencies. As trial designs evolve to include adaptive structures, multiple treatment arms, and longitudinal assessments, there is an increasing demand for formal methods that can represent the full temporal structure of clinical trial execution [5].

One of the challenges in clinical trial informatics is the lack of a formal representation framework for trial protocol specifications. Basic protocol components such as treatment windows, observation periods, washout phases, and outcome evaluations are usually described informally or in free-text, making them difficult to reuse and interpret computationally. This limitation impairs the ability to simulate trials, align patient trajectories, validate protocol logic, or integrate information across studies [34].

Temporal logic provides a formal framework for reasoning about time, enabling the representation of temporal facts and relationships such as event ordering and interval duration [48]. Foundational work like Allen’s interval-based temporal logic has shaped this field by formalizing temporal relations between intervals, laying the groundwork for advanced temporal inference [40]. The expressive power of temporal logic is particularly valuable in clinical contexts, where complex temporal dependencies must be modeled to simulate trial scenarios and interpret patient narratives [18, 48]. In clinical trials, temporal reasoning is pivotal for structuring protocol logic, modeling event sequences, and enforcing timing constraints [39, 30]. These capabilities support key tasks such as cohort modeling, protocol validation, eligibility screening, and outcome prediction across the trial lifecycle. Recent advances have also integrated temporal logic into clinical natural language processing (NLP), improving the extraction and prediction of temporally grounded events [41]. By incorporating temporal entailment between patient states, these methods enable longitudinal reasoning essential for assessing treatment efficacy and guiding patient care [1, 41].

Despite such developments and potentials, there is a lack of logical-framework with demonstrated capability and translatability in representing clinical trial protocols with temporal and semantic precision and the ability to support automated reasoning, leverage data across studies and sites, or simulate trial execution under varying assumptions using Real-World Data [22, 35, 49]. This paper introduces a framework for formalized representation of clinical trials using Temporal Ensemble Logic (TEL [48]). TEL incorporates metricized modal operators, such as “always until *t*” (${◻}_{t}$) and “possibly until *t*” (${♢}_{t}$), where *t* is a time-length parameter, to offer a logical framework for capturing phenotypes in biomedicine. TEL is more expressive in syntax than classical linear temporal logic (LTL) while maintaining the simplicity of semantic structures.

The attributes of TEL are exploited in this paper to formally represent not only individual clinical trial components, but also the timing and sequential dependencies of these components as a whole. We demonstrate that TEL can facilitate formal modeling of key elements including eligibility criteria, baseline visits, interventions, follow-up visits, and outcome assessments. It supports both individual-level and cohort-level reasoning, making it well-suited for clinical trial simulation. Our modeling strategy uses atomic propositions formulated from a domain-specific ontology constructed from standardized biomedical vocabularies such as SNOMED CT [10], ICD-9/10-CM [16], CPT [12], RxNorm [28], and LOINC [25]. Trial participants’ electronic medical records serve as models, over the domain of positive integers $N^{+}$ which represent days (typically the finest granularity in clinical trial protocols), weeks, or months.

The main contributions of this study are as follows: 1) we introduce a modeling strategy to capture clinical trial protocol components using TEL, providing a logical specification of temporal dependencies in clinical trial protocols; 2) we develop the AD Clinical Trial Ontology (ADCTO), a lightweight, domain-specific ontology aligned with biomedical standards to codify semantic entities used in TEL for this purpose, and 3) we prototype a simulation system with interfaces that represent trial specifications as TEL formulas and executes them against Real-World Data (RWD) to support protocol validation and virtual execution using the model-checking paradigm.

The remainder of this paper is organized as follows: Section 2 introduces the formal foundation of TEL. Section 3 describes two major categories of trial simulations. Section 4 presents our modeling of clinical trials. Section 5 details the TEL-based logical formalization of clinical trial components. Section 6 reports on the implementation, ontology development, and simulation system.

## 2 Preliminaries

### 2.1 Temporal Ensemble Logic

In modeling clinical trials, we consider the time domain of positive integers $N^{+}$, which represent days lapsed from the start of a clinical trial.

The basic construct of TEL [48] includes two types of terms: integer terms over $N^{+}$, and logical formulas. Integer terms *s, t, u, v*, … consist of constants *a, b, c*, …, variables *x, y, z*, … (from a set Var), and the addition of these terms (*s* + *t*). We write Term for the set of all such integer terms. Logical formulas φ consist of primitive propositions *p*, indexed formulas $\varphi_{t}$, Boolean connectors (∧, ¬), time-indexed modalities ${◻}_{t}\varphi$ and ${♢}_{t}\varphi$, and first-order quantification over variables in Var.

In Backus–Naur form (BNF) notation, we have s,t::=a|x|(s+t), where *a* is an integer constant, x∈Var, and (*s* + *t*) represent the addition of two integer terms.

For TEL formula, we have, with *t* for integer term and *x* for variable over $N^{+}$:

$$\varphi ,\psi ::=p\left|\varphi_{t}\right|\neg \varphi |\varphi \wedge \psi |\varphi \vee \psi \left|{◻}_{t}\varphi \right|{♢}_{t}\varphi |\forall x\varphi |\exists x\varphi .$$

Atomic propositions *p* come from a pre-defined set Prop, a set of clinical codes and ontological terms according to the conceptual correspondence provided in Table 1.

To specify the semantics of the above formulas, we define an interpretation as a function $\alpha :N^{+}\to 2^{\mathrm{Prop}}$, which determines, at each time-point $i\in N^{+}$, a subset of atomic propositions that are assigned true. We can use the notation of formal languages to describe such interpretations. The alphabet is $\Sigma \left(=2^{\mathrm{Prop}}\right)$, to account for all possible truth-status of each proposition in Prop at a given time. Following standard formal language convention, $\Sigma^{*}$ represents the set of all finite words over Σ, $\Sigma^{+}$ represents the set of all no-empty finite words over Σ, and $\Sigma^{\omega }$ represents all *ω-words* over Σ. Therefore, we can write *α* in the form of an *ω*-word σ[1]σ[2] …, with σ[i]∈Σ being the *i*th letter of *α* for all *i* ≥ 1.

▶ **Definition 1.** *We define, given an ω-word α* = *σ*[1]*σ*[2] … *over* Σ *and any i* ≥ 1,

(α,i)⊨pifp∈σ[i];

$$\left(\alpha ,i\right)⊨\varphi_{t}\;\text{if}\;\left(\alpha ,i+t\right)⊨\varphi ;$$

(α,i)⊨¬φif(α,i)⊭φ;

(α,i)⊨ϕ∧ψif(α,i)⊨φand(α,i)⊨ψ;

(α,i)⊨ϕ∨ψif(α,i)⊨ψor(α,i)⊨ψ;

$$\left(\alpha ,i\right)⊨{□}_{t}\varphi \;\text{if}\left(\alpha ,j\right)⊨\varphi \;\text{for all}\;j\;\text{with}\;i\leq j<\;i+t;$$

$$\left(\alpha ,i\right)⊨{♢}_{t}\varphi \;\text{if}\left(\alpha ,j\right)\;⊨\;\varphi \;\text{for some}\;j\;\text{with}\;i\leq j<i+t;$$

(α,i)⊨∀xφif for allk≥1,(α,i)⊨φ[x:=k].

(α,i)⊨∃xφif there existsk≥1,(α,i)⊨φ[x:=k],

*where* φ[x:=k] *is the standard syntactic convention for the formula obtained by replacing all free occurrences of variable x in* φ *by constant k*.

### 2.2 Conceptual Correspondence for Trial Modeling

Clinical trials involve complex temporal dependencies between interventions, patient responses, and outcomes, requiring a formal approach to temporal reasoning. Existing logical frameworks in computer science and biomedicine either lack expressiveness for capturing trial dynamics or impose syntactic constraints that limit their applicability to real-world trial scenarios.

To make the technical motivations and design decisions more intuitive, we begin with an illustrative example. This example is intended to highlight the practical needs and conceptual foundations for combining first-order and modal logic primitives in the representation of biological phenotypes. By grounding the discussion in concrete use cases, we aim to clarify the expressive requirements that drive our modeling strategy.

▶ **Example 2.** For an Alzheimer’s Disease (AD) clinical trial of demonstrating the efficacy of at least one dose of a medicine (H3 receptor antagonist) in comparison to placebo on cognitive performance in patients with mild to moderate AD [44], TEL provides a formal structure for representing its constraints and event relationships. The key components of the protocol can be expressed as follows:

$$\exists x\exists i\left[S_{x}\wedge I_{x+i}\wedge {\left({♢}_{4w}B\right)}_{\left(x+i\right)}\wedge {\left({◻}_{24w}T\right)}_{x+i+4w}\wedge {\left({♢}_{10w}F\right)}_{x+i+28w}\wedge {\left({♢}_{1w}O\right)}_{x+i+38w}\right]$$

where the start date *x* corresponds to the date of informed consent (*S*). The index event (*I*) is defined as the cognitive evaluations and biomarker analyses *i* days after consent. Baseline visit (*B*) is conducted within four weeks (4w) prior to intervention. The intervention phase (*T*) involves the administration of a medicine or placebo over 24 weeks (24w). Follow-up visits (*F*) are scheduled to monitor safety and efficacy parameters for up to 10 weeks (10w). The outcome assessment (*O*) occurs at week 38 (38w), evaluating changes in cognitive function from baseline.

### 2.3 Related Work: Temporal Logics for Clinical Trials

Many existing temporal logics have been developed to express different types of temporal relationships, each with its own strengths and trade-offs. LTL [33] captures linear sequences of states to express event orders. Based on LTL, Metric Temporal Logic (MTL) [29] adds explicit timing bounds to temporal operators. Interval Temporal Logic (ITL) [9, 14] focuses on intervals rather than points, capturing relationships. Despite the extensive development of temporal logic frameworks, their practical application in clinical trial specification remains limited. Giordano et al. modeled stroke guidelines as concurrent processes in LTL and verified them with SPIN [13], while Sanati et al. introduced a MTL-based Description Logics for formulating eligibility criteria for clinical trials [2]. Shankar et al. [39] developed a formal ontology to encode temporal constraints in clinical trials, allowing precise specification of timeline requirements (e.g., “lab test 2 must occur within 7 days after dose 1”) using ITL. CNTRO [42, 43] encodes logical and temporal constraints directly within the structure of the Web Ontology Language (OWL). The Time Event Ontology (TEO) proposed by Li et al. [21] extends CNTRO by harmonizing Allen’s interval algebra with a suite of basic time relations, although it lacks a language of logic for modeling trials.

LTL can express sequential temporal dependencies and comes with mature automated tools, but it has limited expressiveness. It cannot capture complex interval constraints such as “observe within a specified time window”. MTL can express time-bounded constraints such as visit windows or dosing intervals, but cannot directly relate distinct times or intervals. It lacks the ability to express dependencies such as “dose two administered exactly 14 days after dose one, followed by a visit 7 days later.” ITL can model observation windows and treatments with interval-based semantics (e.g., “treatment continues during observation window”) but it becomes overly complex and lacks cross-timeline reference capability without additional constructs. Table 2 summarizes these observations.

The use of TEL for trial modeling brings the following advantages. TEL has already been demonstrated as a natural but minimalistic logical framework with potential as an attractive approach to formalizing phenotypes in biomedicine [48]. Using the model-checking paradigm, TEL can support both point-based and interval-based temporal constructs. TEL is highly expressive in modeling temporal properties, but restricted in aspects not essential to targeted applications. The scope of first-order quantifiers for TEL is limited to unary predicates derived from an existing formula, rather than over independently given predicates. As can be seen in subsequent developments, the specific fragment of TEL used for this study does not involve the ∀ quantifier. Although it is beyond the scope of the current paper to address theoretical developments in decidability and algorithmic complexity results for specific fragments of TEL (given its general undecidability [48]), our experimental results show practical feasibility in reasonable settings.

## 3 Clinical Trial Simulation (CTS)

Clinical trials serve as the cornerstone of medical research, providing rigorous assessments of the safety and efficacy of novel interventions. However, traditional clinical trial methodologies have several challenges, including ethical considerations, patient recruitment difficulties, and potential confounding variables. Advanced simulation-based approaches have been developed to address these limitations, leveraging real-world data and computational modeling to enhance trial design and execution [4]. This paper examines two key types of clinical trial simulations [19]: Self-Controlled Case Series [31] and Eligibility Cohort Building [11].

### 3.1 Self-Controlled Case Series (SCCS)

The Self-Controlled Case Series (SCCS) method is a widely used design for evaluating the association between medical interventions and adverse events. By treating individuals as their own controls, SCCS effectively mitigates confounding from fixed patient-level characteristics such as genetics and lifestyle [46]. In this simulation-based framework, longitudinal health records are used to construct individual timelines of intervention exposure and subsequent adverse events. A predefined risk window is specified following the intervention, and the frequency of events during this period is compared to baseline periods within the same individual using statistical modeling.

For example, a recent study investigating thrombosis risk after COVID-19 vaccination applied SCCS by extracting temporal sequences of vaccination dates and thrombotic events from electronic health records [17]. By comparing the incidence of events before and after vaccination within each individual, the study reduced between-subject variability and provided robust evidence of causal relationships.

### 3.2 Eligibility Cohort Specification

Patient recruitment is a persistent bottleneck in clinical trial implementation. Eligibility Cohort Building Simulations offer a data-driven strategy to improve recruitment efficiency by leveraging EHR data to model real-world patient populations [36]. In this approach, researchers first define trial eligibility criteria such as age range, treatment history, and comorbidities, and apply them to EHR datasets to construct a virtual cohort. Computational models estimate the number of patients who meet the criteria and allow iterative refinement by adjusting thresholds or conditions to assess their impact on cohort size and diversity [20]. This simulation process enables the design of recruitment strategies that are both scientifically rigorous and operationally feasible.

For example, a Phase III clinical trial investigating a novel therapeutic for hormone receptor-positive breast cancer used this method to address recruitment delays. By simulating eligibility criteria including age between 30 and 75 years, prior chemotherapy, and absence of major cardiovascular conditions on a real-world breast cancer dataset, investigators identified constraints that limited enrollment. Adjusting these parameters, such as slightly expanding the age range, helped increase the candidate pool while preserving trial integrity [26].

## 4 Structure of Clinical Trial Specification

### 4.1 Individual Timeline-Based Structure of CTS

#### Start Event (Initialization).

The start event denotes the earliest temporal reference point preceding trial enrollment. It may represent initial patient contact, the commencement of a data collection window, or the initiation of eligibility surveillance. Although not part of the trial intervention itself, this event serves as a critical anchor for modeling retrospective observation periods, washout phases, or baseline covariate collection from real-world data sources such as electronic health records (EHRs) or insurance claims.

#### Index Event (Trial Anchor Point).

The index event marks the initiation of the trial timeline for each participant. Defined via clinical markers or diagnostic confirmation, this event anchors the temporal alignment of all subsequent protocol components. It facilitates consistent modeling of participant trajectories across eligibility evaluation, randomization, and treatment administration phases.

#### Observation Window (Pre-Index Interval).

The interval between the start and index events constitutes the observation window, during which baseline covariates, prior exposures, or exclusionary conditions are assessed. This period may include look-back or washout durations essential for contextualizing eligibility and anchoring the index event.

#### Eligibility Criteria (Inclusion and Exclusion).

Eligibility is determined based on defined inclusion and exclusion criteria. Inclusion criteria specify characteristics that must be present (e.g., age range, disease severity), while exclusion criteria identify disqualifying conditions (e.g., contraindications, comorbidities). These filters are applied either before or at the index to ensure clinical appropriateness and cohort homogeneity.

#### Baseline Visit (Pre-Intervention Assessments).

Participants meeting eligibility requirements undergo a baseline visit for comprehensive pre-intervention evaluation. This includes the collection of demographic data, clinical measurements, and relevant biomarkers, which serve as reference points for outcome comparison and guide treatment group allocation.

#### Intervention Phase (Treatment Administration).

The intervention phase involves the administration of assigned treatments such as experimental therapies, standard care, or placebos, initiated after the index event. This phase captures the protocol-defined exposure period and may incorporate adherence, dose variability, or pharmacokinetic modeling.

#### Follow-Up Visits (Post-Treatment Monitoring).

Follow-up visits are conducted at scheduled intervals following the intervention phase to monitor clinical outcomes, detect adverse events, and track longitudinal changes in patient status. This component accounts for dropout risk, adherence behavior, and the timing of outcome manifestation.

#### Outcome Assessment (End Event).

The final phase of the trial involves assessing predefined primary and secondary endpoints, including clinical efficacy, safety profiles, or composite outcomes. Outcome evaluation is conducted after the full observation period has elapsed or upon reaching specific clinical milestones, enabling rigorous analysis of treatment effects.

### 4.2 Cohort-Based Structure of CTS

The Individual Timeline-Based CTS captures the temporal dynamics of clinical events at the patient level, supporting analyses of time-dependent phenomena such as treatment onset and disease progression. While effective for intra-individual variability and temporal causality, it is less suited for between-group comparisons. In contrast, the Cohort-Based CTS organizes participants into predefined groups based on treatment or baseline characteristics, simulating longitudinal outcomes to assess efficacy, safety, event incidence, and dropout rates. This structure, aligned with parallel-arm trial designs, aids trial feasibility, sample size estimation, and recruitment planning. Cohorts are defined by Base Criteria (shared attributes), Case Criteria (presence of key exposures), and Control Criteria (absence of exposures), enabling robust comparative analyses and accounting for real-world complexities such as time-to-event variability, adherence, and attrition.

## 5 Logical Representation of CTS using Temporal Ensemble Logic

### 5.1 TEL-Based Formalization of Timeline-based CTS

This section formalizes a timeline-based CTS using the TEL framework. The model is anchored by a start event (*S*) and an index event (*I*), such as a diagnosis or lab finding, which serves as the temporal reference for aligning all other trial components. Between these events lies the observation window (*W*), used to assess baseline covariates and eligibility status. Eligibility criteria (*E*) may span both pre- and post-index periods. The baseline visit (*B*) captures pre-intervention assessments. The intervention phase (*T*) begins after the index event, followed by post-treatment follow-up visits (*F*) to monitor outcomes and safety. Outcome assessment (*O*) occurs within a bounded period at the end of the trial. TEL constraints are defined with modal operators to precisely capture temporal relationships.

- **Start Event**: The TEL expression for the start event occurring at time *x* is: $\exists x\left(S_{x}\right)$.
- **Index Event**: The TEL expression for the index event occurring at time *x* + *i* is given by: $\exists x\exists i\left(S_{x}\wedge I_{x+i}\right)$. The *x* + *i* subscript ensures that Index event can occur only after the Start Event. This represents the key modeling strategy of using distinct terms to represent time dependencies, rather than using explicit comparison such as *x* < *y* in other approaches.
- **Observation Window**: The observation window defines the time interval between the start event and the index event, within which prior clinical events must occur. The corresponding TEL expression is given by: $\exists x\exists i\left[S_{x}\wedge I_{x+i}\wedge {\left({♢}_{i}W\right)}_{x}\right]$.
- **Baseline Visit**: The TEL expression for the baseline visit *B* occurs within *b* units after the index event is given by: $\exists x\exists i\exists b\left[S_{x}\wedge I_{x+i}\wedge {\left({♢}_{i}W\right)}_{x}\wedge {\left({♢}_{b}B\right)}_{x+i}\right]$.
- **Intervention Phase**: The TEL expression for the intervention phase *T* occurring within *t* units post-baseline is given by:
$$\exists x\exists i\exists b\exists t\left[S_{x}\wedge I_{x+i}\wedge {\left({♢}_{i}W\right)}_{x}\wedge {\left({♢}_{b}B\right)}_{x+i}\wedge {\left({♢}_{t}T\right)}_{x+i+b}\right].$$
- **Follow-up visits**: The TEL expression for the follow-up visit *F* occurring within *f* units after the intervention phase is given by:
$$\exists x\exists i\exists b\exists t\exists f\left[S_{x}\wedge I_{x+i}\wedge {\left({♢}_{i}W\right)}_{x}\wedge {\left({♢}_{b}B\right)}_{x+i}\wedge {\left({♢}_{t}T\right)}_{x+i+b}\wedge {\left({♢}_{f}F\right)}_{x+i+b+t}\right].$$
- **Outcome Assessment**: The TEL expression for the outcome assessment *O* occurring within *o* units after the follow-up visit is given by:
$$\exists x\exists i\exists b\exists t\exists f\exists o\left[S_{x}\wedge I_{x+i}\wedge {\left({♢}_{i}W\right)}_{x}\wedge {\left({♢}_{b}B\right)}_{x+i}\wedge {\left({♢}_{t}T\right)}_{x+i+b}\wedge {\left({♢}_{f}F\right)}_{x+i+b+t}\wedge {\left({♢}_{o}O\right)}_{x+i+b+t+f}\right].$$
- **Eligibility Criteria**: Eligibility Criteria define the conditions under which a participant is deemed suitable for enrollment in the clinical trial.
  - **Inclusion Criteria (***IC***)**: which specify characteristics that must be present for a participant to qualify. The TEL expression is given by:
$$\exists x\exists i\exists c_{1}\exists c_{2}\left[S_{x}\wedge I_{x+i}\wedge {\left({♢}_{c_{1}}IC\right)}_{x}\vee {\left({♢}_{c_{2}}IC\right)}_{x+i}\right].$$
  - **Exclusion Criteria (***EX***)**, which specify conditions that, if present, disqualify a participant from participation. The TEL expression is given by:
$$\exists x\exists i\exists e_{1}\exists e_{2}\left[S_{x}\wedge I_{x+i}\wedge {\left({◻}_{e_{1}}\neg EX\right)}_{x}\wedge {\left({◻}_{e_{2}}\neg EX\right)}_{x+i}\right].$$

The full TEL expression representing the clinical trial structure temporally anchored on the start event and index event, and encoding the logical and temporal constraints across eligibility, baseline, treatment, follow-up, and outcome phases, is defined as follows:

$$\exists x\exists i\exists b\exists t\exists f\exists o\exists f\exists c_{1}\exists c_{2}\exists e_{1}\exists e_{2}\left[S_{x}\wedge I_{x+i}\wedge {\left({♢}_{i}W\right)}_{x}\wedge {\left({♢}_{b}B\right)}_{x+i}\wedge {\left({♢}_{t}T\right)}_{x+i+b}\wedge {\left({♢}_{f}F\right)}_{x+i+b+t}\wedge \right.\;\;\left.{\left({♢}_{o}O\right)}_{x+i+b+t+f}\wedge \left({\left({♢}_{c_{1}}IC\right)}_{x}\vee {\left({♢}_{c_{2}}IC\right)}_{x+i}\right)\wedge \left({\left({◻}_{e_{1}}\neg EX\right)}_{x}\wedge {\left({◻}_{e_{2}}\neg EX\right)}_{x+i}\right)\right].$$

### 5.2 TEL-Based Formalization of Cohort-based CTS

The Cohort-Based CTS models trial participants as belonging to logically distinct subpopulations, defined through a combination of base eligibility and group-specific cohort criteria. Each individual in the simulation is first evaluated against the Base Criteria to determine inclusion in the eligible study population. Then, individuals are assigned to mutually exclusive groups according to whether they satisfy the Case Criteria or Control Criteria.

- **Base Criteria**: All individuals must meet the base criteria within a defined observation window starting at the reference time $t_{0}$. These criteria typically include basic demographic filters, the length of the observation period, and the exclusion of confounders. The TEL expression is: $\exists t_{0}({\mathrm{Base}}_{t_{0}})$.
- **Case Criteria**: Individuals who meet the case criteria are assigned to the case cohort. These criteria usually capture exposure to an intervention, diagnosis of a condition, or other qualifying events. The case criteria must be met after satisfying the base criteria. The TEL expression is: $\exists t_{0}\exists t_{1}[{\mathrm{Base}}_{t_{0}}\wedge {\left({♢}_{t_{1}}\text{Case}\right)}_{t_{0}}]$.
- **Control Criteria**: Individuals who meet the control criteria are assigned to the control cohort. These criteria typically indicate the absence of the exposure or condition that defines the case group. The assignment of control also follows the base criterion. The TEL expression is: $\exists t_{0}\exists t_{2}[{\mathrm{Base}}_{t_{0}}\wedge {\left({♢}_{t_{2}}\text{Control}\right)}_{t_{0}}]$.

### 5.3 Model-Checking of Clinical Trial Specifications

Formally, the model-checking [6, 45] process seeks to determine whether a patient record *α* satisfies a given clinical trial specification *φ*, as defined in TEL. That is, we aim to verify whether there exists a time point *i* ≥ 1 such that (α,i)⊨φ, under the TEL semantics. This generalizes beyond traditional initial-time evaluation by enabling satisfaction to be checked at any point along a patient’s longitudinal record, allowing for alignment of protocol logic with varying index events. The language defined by a TEL formula, denoted ℒ(φ), consists of all *ω*-words (i.e., patient records) that satisfy the formula at some valid time index. This capability supports temporal abstraction and flexible anchoring of eligibility and trial components in real-world datasets. TEL-based model-checking provides a scalable and precise method for aligning structured clinical data with complex temporal protocol logic, improving the interpretability, consistency, and reproducibility of virtual clinical trials.

## 6 Case Study

To evaluate and demonstrate the practical utility of our TEL-based formalization framework, we applied it to the domain of AD clinical trials. AD trials present complex temporal structures and heterogeneous eligibility criteria, making them a representative use case for formal modeling. We developed two resources: (1) the AD Clinical Trial Ontology (ADCTO), a lightweight ontology that captures core concepts from AD-related trial protocols, and (2) a logic-based clinical trial simulation system that operationalizes TEL for temporally-aware trial design and execution. These tools enable precise specification, validation, and simulation of AD clinical trials grounded in real-world data.

### 6.1 AD Clinical Trial Ontology

We developed the AD Clinical Trial Ontology (ADCTO) to represent key elements frequently encountered in the eligibility criteria of Alzheimer’s disease (AD) clinical trials, using data from ClinicalTrials.gov. The ontology encompasses a comprehensive range of categories, including Disease, Medication, Diagnostic Test, Procedure, Rating Criteria, Social Determinants of Health, and Fertility. Each concept was mapped to the Unified Medical Language System (UMLS) with assigned Concept Unique Identifiers (CUIs) and annotated with Observational Health Data Sciences and Informatics (OHDSI) Athena identifiers. We enriched ADCTO with annotations from resources such as the National Library of Medicine (NLM) Value Set Authority Center, the National Drug Code (NDC) directory, UMLS Terminology Services, Codify by the American Academy of Professional Coders (AAPC), and the Phenotype KnowledgeBase (PheKB). To construct a coherent hierarchy, we integrated established biomedical ontologies and classification systems, including the Disease Ontology for disease concepts and DrugBank and the Anatomical Therapeutic Chemical (ATC) classification system for medications, with refinements informed by domain experts to ensure semantic precision and relevance.

Compared to existing ontologies, ADCTO maintains a lightweight structure, with 274 classes, 278 logical axioms, and 284 declaration axioms. To evaluate its adequacy, we achieved a recall of 0.63 (the proportion of ontology-mapped elements relative to all extracted eligibility elements) and an average term frequency–inverse document frequency (TF-IDF) score of 3.91, indicating ADCTO’s effectiveness in capturing the essential elements of AD eligibility criteria in a compact and computationally efficient structure.

### 6.2 AD Clinical Trial Simulation System

We developed the AD Clinical Trial Simulation System, a logic-based platform for designing and evaluating virtual clinical trials using real-world data. Built on the ADCTO, the system provides a formally defined and semantically consistent vocabulary to represent key trial components, including event types, eligibility criteria, interventions, outcomes, and their temporal dependencies. Users interact with a web-based interface that offers ontology-aligned dropdown menus, enabling the configuration of trial components without the need for manual coding. These selections are automatically compiled into formal representations, allowing for precise and interpretable modeling of trial logic. The simulation engine leverages TEL to capture and enforce time-dependent relationships among clinical events. All components are temporally anchored to a defined index event, enabling precise sequencing of trial activities. The system supports fixed and flexible timelines and allows retrospective simulations using observational data. It is designed to bridge the gap between descriptions of natural language protocols and formal computational models. The user interface reflects the core vision of the system, which is to democratize formal trial design by making logical and ontological structures accessible to clinical researchers without requiring expertise in logic or programming.

**Figure 1:**
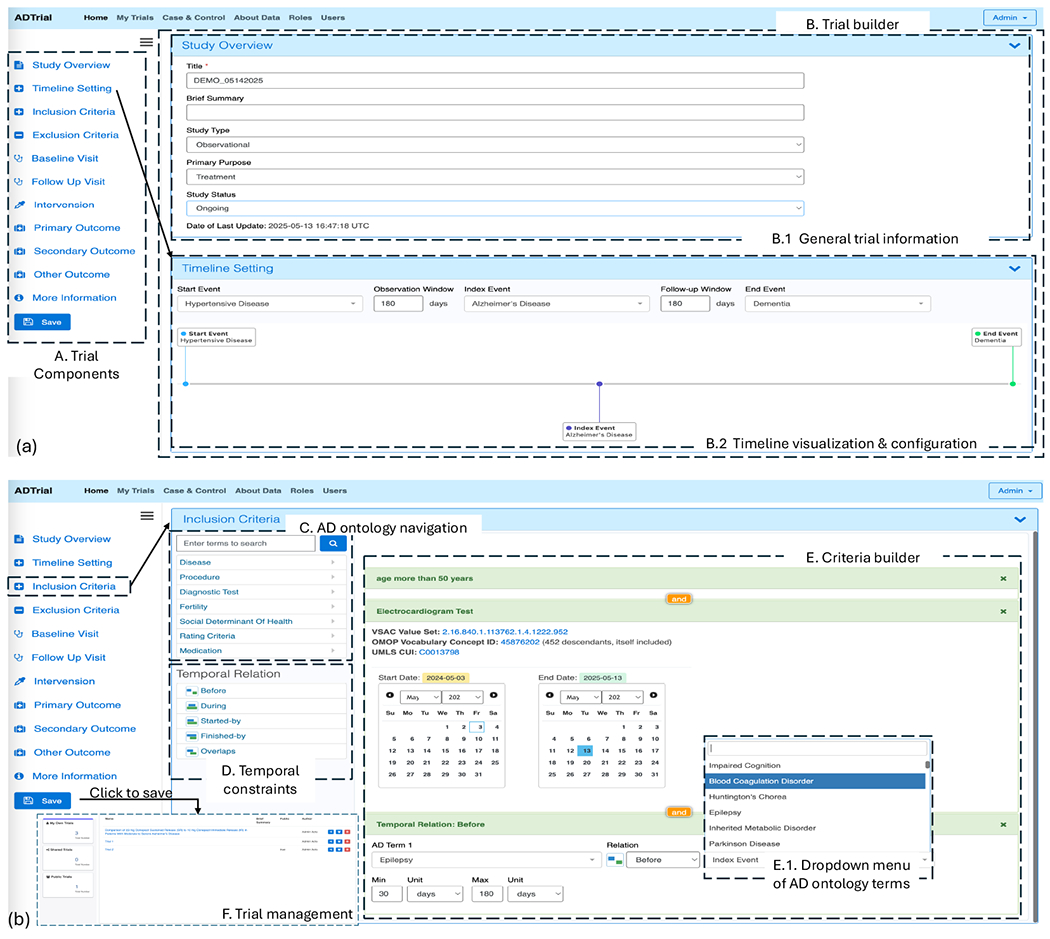
The interface of the AD clinical trial simulation system.

Figure 1 illustrates the user interface of our system. Figure 1.(a).A shows the components of a clinical trial and allows users to click on each element to begin configuring it. Figure 1.(a).B showcases the trial builder, with subpanels B.1 and B.2 displaying interfaces for entering trial information and configuring timeline parameters. Figure 1(b) illustrates the configuration interface for specifying Inclusion Criteria, which serves as a representative example; similar interfaces are employed across other trial components to ensure consistency and usability. Figure 1.(b).C presents the ontology navigation interface, enabling users to explore standardized ADCTO terms. Figure 1.(b).D and E highlight the specification of temporal constraints and eligibility criteria, with E.1 featuring a dropdown menu populated by ontology-aligned terms. Finally, Figure 1.(b).F depicts the trial management interface, where users can review and save configured trials. Configured trials are automatically translated into formal TEL expressions and executed against real-world EHR datasets to simulate trial behavior and validate protocol feasibility. This enables researchers to assess cohort sizes, timing constraints, and outcome trajectories in silico. The system was implemented using Ruby on Rails [15] with a Model-View-Controller (MVC) architecture and used MongoDB [3] as the backend database to support scalable, document-based storage of evolving trial representations and large-scale simulations.

The interface serves as an authoring tool and compilation engine, generating formal TEL models from high-level trial designs. Each session outputs a reusable TEL expression anchored by start and index events, enriched with modal and quantified constraints. These specifications support downstream tasks such as simulation, model-checking, and validation against EHR or registry data. The platform enforces an ontology-driven workflow that enhances semantic clarity, reduces variability, and promotes reuse across studies. Aligned with standardized vocabularies, it enables integration with external data models and supports automated reasoning. Designed for longitudinal datasets, the system facilitates rapid prototyping, cohort selection, and eligibility screening. Each simulation is saved as a machine-readable specification that adheres to FAIR (Findable, Accessible, Interoperable, Reusable) data principles, supporting reproducibility, version control, and collaboration across institutions.

## 7 Discussion

The simulation system implements a formal, TEL-based approach for specifying and simulating clinical trials, supporting ontology-driven configuration and automatic translation of protocol elements into logical expressions. The system represents an early-stage prototype designed to establish the feasibility of logic-based trial modeling and virtual execution. Development priorities include extending the reasoning engine, improving user interface interactivity, supporting integration with large-scale observational data platforms, and enabling export of TEL specifications in interoperable formats aligned with common data models. These enhancements will allow the platform to support complex protocol designs and to enable real-time simulation of cohort definitions and outcome trajectories. The current system implements a pipeline that maps user interface inputs directly to TEL formulas. Trial designers specify protocol elements using dropdown menus populated from ADCTO, including time constraints and event types. These inputs are compiled into formal TEL expressions through structured templates that preserve both syntactic correctness and semantic alignment. Patient medical records are represented as temporally indexed sequences of coded events, enabling the TEL engine to evaluate whether a given specification is satisfied at any admissible index point in the record.

TEL is undecidable in its general form [48]; however, the fragment used in this study avoids intractable features such as unrestricted quantifier alternation and deeply nested temporal operators. It relies on existential quantification and time-bounded modal constructs, allowing finite evaluation over RWD. We have developed and preliminarily validated algorithms for this purpose, with several manuscripts in preparation detailing their design, implementation, and performance benchmarking. These works will also introduce interval-based extensions to model durations between temporally disjoint events. Formal characterization of the fragment’s decidability and complexity remains a key direction for ensuring scalability.

In its current form, TEL supports point-based reasoning with relative offsets and can explicitly model upper bounds on absolute time intervals between events. Using the modal operators ${♢}_{t}$ (diamond) and ${◻}_{t}$ (box) with time parameters, TEL can precisely define both lower and upper bounds for the occurrence of events. For example, ${◻}_{t}\varphi$ ensures that a condition *φ* holds continuously for a duration of *t* time units, while ${♢}_{t}\varphi$ guarantees that *φ* becomes true at some point within *t* units. These constructs provide direct control over temporal constraints, which is critical in clinical trial protocols where the timing of events, such as symptom onset, dosing intervals, or outcome assessments, is clinically significant. This expressiveness allows TEL to model temporally bounded windows of clinical relevance and supports fine-grained specification of interval-based requirements.

TEL has focused on clinical trial modeling, but there is a range of related efforts that address temporal representations in healthcare processes, such as clinical guidelines, care pathways, and decision-intensive workflows. Relevant work includes temporal workflow models for clinical pathways [7], decision modeling frameworks for chronic care management [8], and Business Process Model and Notation (BPMN)-based systems [24] for perioperative processes. Other studies have explored mobile delivery of guideline-based decision support [38], ontology-based search and reasoning over clinical protocols [27], and logic-based eligibility verification for trials [23]. Metric and interval temporal logic approaches have been applied to model timing constraints in clinical practice guidelines [37, 47]. Our approach complements these efforts by offering a logic tailored for patient-level temporal event alignment with computable representations integrated into simulation engines and ontology-driven user interfaces.

Finally, TEL captures temporal and structural dependencies, but clinical trials often involve normative constraints. Deontic logic provides a formal foundation for representing requirements such as obligations, prohibitions, and permissions. Integrating deontic logic into the TEL framework may enable richer modeling of regulatory rules, protocol compliance, and adaptive trial conditions. This direction will enhance the system’s ability to represent real-world clinical scenarios that involve both temporal precision and rule-governed behaviors.

## 8 Conclusion

This study presents a TEL-based framework for modeling clinical trials through precise, formal logic representations of temporal relationships among clinical events. It addresses key limitations in current trial design, including the lack of formal temporal reasoning and unstructured protocol specifications. We developed a logic-based simulation system and the ADCT Ontology to standardize trial components and support formal reasoning. The system includes an ontology-driven user interface that enables domain experts to configure trials without coding, facilitating reproducible virtual trial design, feasibility analysis, and cohort simulation. The methodology generalizes to other domains requiring semantically rigorous and temporally aligned trial models. TEL has limited direct support from widely available verification or modeling platforms, and future work will focus on developing dedicated implementations to enhance its adoption and usability.

## Figures and Tables

**Table 1:** Correspondence Between Clinical Trial Components and TEL Elements.

Clinical Trial Entities	Logical Constructs	Logical Forms
Controlled vocabularies, ontology terms	Atomic propositions	Prop
Temporal relationships	Modal and logical operators	${◻}_{t}$, ${♢}_{t}$, ∧, ∃
Electronic medical records	Semantic models	σ[1]σ[2]⋯
Start Event, Index Event, Inclusion Criteria, Exclusion Criteria, Observation Window, Baseline Visit, Intervention Phase, Follow-Up Visits, Outcome Assessment, Base Criteria, Case Criteria, Control Criteria	TEL formulas	*S, I, IC, EC, W, B, T, F, O, Base, Case, Control*
Trial execution	Model-checking	(α,i)⊨φ

**Table 2:** Logic-based Modeling for Clinical Trials.

Logic	Constructs	Expressiveness	Clinical Trial Application	Modeled Component
LTL	Point-based Decidable	Limited	[13]	Entire trial
MTL	Point-based Decidable (restricted cases)	Limited	[2]	Eligibility criteria
ITL	Interval-based Decidable (restricted cases)	Lack point-based references	[21, 39, 42]	Entire trial
**TEL**	**Point-based, limited First-order Decidability for used fragment open**	**Good fit for purpose (integrative, interval and point, first-order)**	**This study**	**Entire trial**
